# Supplementary material for: Exercise-Based Interventions to Enhance Long-Term Sustainability of Physical Activity in Older Adults: A Systematic Review and Meta-Analysis of Randomized Clinical Trials
Source: Int J Environ Res Public Health. 2019 Jul 15;16(14):2527. doi: 10.3390/ijerph16142527 (PMC6678490; doi:10.3390/ijerph16142527)
Supplement: Supplementary file 1 [file ijerph-16-02527-s001.zip › ijerph-528093/Supplementary_Table_S1_search_strategy.docx]

**Supplementary Table S1**

**MEDLINE (PubMed)**

January 2018

#1        “Aging”[MeSH Terms] OR “Nursing Homes”[MeSH] OR “Long-term care” [MeSH] OR “Caregivers” [MeSH] OR “Homebound Persons”[MeSH] OR “Home care services”[MeSH] OR old[Title/Abstract] OR olds[Title/Abstract] OR senior[Title/Abstract] OR seniors[Title/Abstract] OR ageing[Title/Abstract] OR aging[Title/Abstract] OR aged[Title/Abstract] OR nursing home*[Title/Abstract] OR community dwelling[Title/Abstract] OR care home*[Title/Abstract] OR carer[Title/Abstract] OR carers[Title/Abstract] OR long-term care[Title/Abstract] OR caregiver[Title/Abstract] OR care giver[Title/Abstract] OR caregivers[Title/Abstract] OR care givers[Title/Abstract] OR homebound[Title/Abstract] OR resident*[Title/Abstract]  1828945

#2        (“Adult”[MeSH] OR “Middle Aged”[MeSH] OR “Young Adult”[MeSH] OR Child[MeSH] OR “Child, Preschool”[MeSH] OR Infant[MeSH] OR “Infant, Newborn”[MeSH] OR “Internship and Residency”[MeSH OR adolescent*[tiab] OR youth*[tiab] OR young[tiab] OR child*[tiab] OR pediatric[tiab] OR paediatric[tiab]) NOT "Aged"[MeSH]        1582329

#3        #1 NOT #2        1482560

#4        "Aged"[MeSH] OR “Geriatrics” [MeSH] OR older[tiab] OR oldest[tiab] OR elder[tiab] OR elderly[tiab] OR elders[tiab] OR eldership[tiab]   2929284

#5        #3 OR #4          3908325

#6        “Exercise”[Majr] 101919

#7        “Exercise Therapy”[Mesh]           39343

#8        "Physical Fitness"[Mesh]  24808

#9        exercise[ti]         92365

#10       physical activity[tiab]      81156

#11       physical train*[ti]            2088

#12       fitness[ti]           15182

#13       aerobic[ti]         15348

walking

#14       #6 OR #7 OR #8 OR #9 OR #10 OR #11 OR #12 OR #13  265270

#15       #5 AND #14      71955

#16       sustain*[tiab]     272669

#17       continued[ti]      4396

#18       continuation[ti]   1902

#19       maintained[ti]    4914

#20       maintenance[ti]  33877

#21       long term[ti]      171647

#22       intensity[ti]         33089

#23       follow up[ti]       84979

#24       #16 OR #17 OR #18 OR #19 OR #20 OR #21 OR #22 OR #23       580225

#25       #15 AND #24    4709

#28       systematic[sb]    328193

#29       #25 AND #28    123

#30       #25 NOT #29    4586

#31       (randomized controlled trial[pt] OR controlled clinical trial[pt] OR randomized[tiab] OR placebo[tiab] OR drug therapy[sh] OR randomly[tiab] OR trial[tiab] OR groups[tiab]) NOT (animals [mh] NOT humans [mh])          3505571

#32       #30 AND #31    2146

#34       #25 NOT (#29 OR #32)  2440

#35       #34 AND 28648951[uid] 0

#36       28648951[uid]    1

#37       longitudinal[tiab] OR cohort*[tiab]           576315

#38       #15 AND #37    7562

#40       longitudinal[ti] OR cohort*[ti]      110398

#41       #15 AND #40    1923

**OVID Embase <1974 to 2017 November 16>**

January 2018

1     aged/ (2671375)

2     older.ti,ab. (476520)

3     elderly.ti,ab. (295651)

4     1 or 2 or 3 (3015661)

5     *exercise/ (103420)

6     exp fitness/ (35889)

7     exercise.ti. (118971)

8     physical activity.ti,ab. (112787)

9     5 or 6 or 7 or 8 (275973)

10     sustain*.ti,ab. (369026)

11     continued.ti. (5147)

12     continuation.ti. (2440)

13     maintained.ti. (5703)

14     maintenance.ti. (43899)

15     long term.ti. (233163)

16     follow up.ti. (117109)

17     10 or 11 or 12 or 13 or 14 or 15 or 16 (737794)

18     4 and 9 and 17 (2692)

19     random:.tw. or placebo:.mp. or double-blind:.tw. (1507141)

20     18 and 19 (777)

**The Cochrane Library <Cochrane Central Register of Controlled Trials : Issue 10 of 12, October 2017>**

January 2018

#1        MeSH descriptor: [Aged] explode all trees           1180

#2        older:ti,ab         29155

#3        elderly:ti,ab       19950

#4        #1 or #2 or #3 45696

#5        MeSH descriptor: [Exercise] explode all trees       19952

#6        MeSH descriptor: [Exercise Therapy] explode all trees      11092

#7        MeSH descriptor: [Physical Fitness] explode all trees        2760

#8        exercise:ti          23253

#9        (physical next activity):ti,ab          12479

#10       #5 or #6 or #7 or #8 or #9        47148

#11       sustain*:ti,ab      25133

#12       continued:ti       472

#13       continuation:ti    517

#14       maintained:ti      546

#15       maintenance:ti   6675

#16       (long next term):ti          21386

#17       (follow next up):ti           12898

#18       #11 or #12 or #13 or #14 or #15 or #16 or #17 62408

#19       #4 and #10 and #18      384
