# Supplementary material for: Exercise-Based Interventions to Enhance Long-Term Sustainability of Physical Activity in Older Adults: A Systematic Review and Meta-Analysis of Randomized Clinical Trials
Source: Int J Environ Res Public Health. 2019 Jul 15;16(14):2527. doi: 10.3390/ijerph16142527 (PMC6678490; doi:10.3390/ijerph16142527)
Supplement: Supplementary file 1 [file ijerph-16-02527-s001.zip › ijerph-528093/Supplementary_Table_S3.docx]

Supplementary Table S3. Description of included studies

| **Study** | **N (% female)** | **Mean age (SD)** | **Inclusion/exclusion criteria** | **Setting** | **PA and physical function outcomes** | **Instruments**  **(*Validated)** | **Time point measures** |
| --- | --- | --- | --- | --- | --- | --- | --- |
| Ståhle et al. 1999 | 109 (20) | IG: 71 (3.9)  CG: 71 (4.7) | Inclusion:  Patients ≥ 65 years admitted to Coronary Care Unit due to and acute coronary event, able to perform a pre-discharge exercise test at a workload ≥ 70W males and ≥ 50W females. For participants with unstable angina a ST depression > 1mm in two adjacent leads had to be documented at the exercise test. | Community | Self-reported PA level. | Six-point scale***** (1 = sedentary and 6 = strenuous exercise comprising at least 3 h/week on such activities as jogging, skiing, tennis, swimming and aerobic training). | Baseline, 3 (end of intervention) and 12 months. |
| Hauer et al. 2003 | 57 (100) | 84.3 (4.4) | Inclusion:  Age ≥ 75 years, recent history of injurious falls, female gender, consent of their orthopaedic surgeon after injurious fall. | Geriatric hospital | 1. Self-reported overall PA with sub scores of homework, leisure time activity, and sportive activity (including walking).  2. General mobility.  3. Maximal gait speed.  4. Balance.  5. Aerobic performance.  6. Lower-limb strength. | 1. Modified Baecke Questionnaire for Older Adults*****.  2. Timed Up and Go Test (TUGT) and Tinetti Performance Oriented Mobility Assessment.  3. Not standardized distance walking speed test.  4. Modified balance test.  5. Stair climbing test.  6. 10-time chair stand test and the step height test. | Baseline, 3 (end of intervention), 6 and 24 months. |
| Beyer et al. 2007 | 65 (100) | IG: 78.6 (5.1).  CG: 77.6 (4.4). | Inclusion:  Home-dwelling women aged 70-90 years, who had suffered a fall that consequently required attention in an emergency room but not hospitalization.  Exclusion:  Fractures of the lower extremities  within the last six months, neurological diseases, inability to understand Danish, and cognitive impairment (MMSE < 24). | Physical therapy gym | 1. Self-reported PA level.  2. Maximal isokinetic and isometric contraction of knee extension and flexion.  3. Maximal isometric trunk extension and flexion force.  4. Habitual and maximal gait speed.  5. Balance. | 1. Self-rated PA level in 4 categories: <2h/w=light PA for less than 2 h/week; 2-4h/w=light PA for 2-4 h/week, >4h/w=light  PA for more than 4 h/week or vigorous PA for 2-4 h/week.  2. Dynamometer.  3. Strain-gauge force transducer.  4. 30-meter walking speed test.  5. Berg Balance Scale. | Baseline, 6 (end of intervention) and 12 months. |
| McAuley et al. 2007 | 174 (72) | 66.70 (5.35) | Inclusion:  Aged 60 to 75 years, sedentary as defined by a lack of regular involvement in exercise during the previous 6 months, healthy and with personal physician's clearance for participation, adequate mental status as assessed by the Pfeiffer Mental Status Questionnaire and willingness to be randomly assigned to either treatment condition. | University setting and local indoor shopping mall.  CG used a gymnasium | Self-report PA level in a 1-week period. | Physical Activity Scale for the Elderly*****. | Baseline, 2 and 5 years. |
| Witham et al. 2007 | 82 (37) | 80.5 (5.0) | Aged 70 years or older with a diagnosis of chronic heart failure. All patients had proven left ventricular systolic dysfunction on echocardiography, radionuclide ventriculography, or contrast ventriculography. | Local heart failure clinic  and medicine for the elderly clinic | 1. PA levels with counts/24h.  2. Aerobic performance. | 1. Stayhealthy RT3 accelerometer*****.  2. 6-minute walk test. | Baseline, 3 (end of intervention), 6 and 12 months. |
| Karinkanta et al. 2009 | 149 (100) | RES: 72.7 (2.5)  BAL: 72.9 (2.3)  COMB: 72.9 (2.2)  CG: 72.0 (2.1) | Inclusion:  70-79-year-old home-dwelling women.  Exclusion:  History of illness contraindicating exercise, history of any illness affecting balance or bones, uncorrected vision problems, taking medication known to affect balance or bone metabolism (for 12 months before the enrolment), intense exercise more than twice a week or the T-score for femoral neck bone mineral density lower than −2.5. | Training center | 1. Weekly self-reported PA level (type, frequency and duration).  2. Maximal isometric contraction of knee extension.  3. Dynamic balance and agility.  4. Self-rated physical function. | 1. PA converted to  MET-h/week.  2. Leg press dynamometer.  3. Standardized figure-of-eight running test.  4. Standardized Finnish Physical Functioning Scale of Rand 36-Item Health Survey. | Baseline, 12 (end of intervention) and 24 months. |
| Rejeski et al. 2009 | 106 (68.9) | Age range 70-89 | Inclusion:  Men and women aged 70-89 years, SPPB summary score less than 10, able to complete the 400-m walk in 15 minutes at baseline, and sedentary (<20 minutes of exercise each week for the past month).  Exclusion:  Major medical or psychiatric condition or MMSE < 21. | University setting | 1. Self-report PA level (duration) across domains, and of different intensities (light, moderate, vigorous) in a 1-week period.  2. Aerobic performance.  3. Physical function (gait speed, balance, lower-limb strength). | 1. CHAMPS PA questionnaire*****.  2. 400-m walk.  3. SPPB. | Baseline, 6, 12 and 36 months. |
| Patel et al. 2013 | 225 (55) | 65-75 years= 78%  ≥76 years= 22% | Inclusion:  Aged ≥ 65 years, speak/write English, able to walk, no contraindications for PA, low PA levels (<150 min/w), reside in community in Auckland (NZ).  Exclusion:  Visual impairment. | Home | Self-report PA level (minutes of PA/week). | Auckland Heart Study PA Questionnaire*****. | Baseline, 3 (end of intervention), and 9 months. |
| Dohrn et al. 2017 | 91 (98) | 75.6 (5.4) | Inclusion:  Community dwelling, aged ≥ 65 years, living in Stockholm county (Sweden), confirmed osteoporosis, impaired balance and fall related concerns.  Exclusion:  Fall related fracture within last year, MMSE < 24, other diseases that impact training ability, inability to walk indoors without aid. | Group setting (not stated) | 1. PA level with counts per minute.  2. Number of sedentary bouts/day.  3. Steps per day. | 1. Actigraph accelerometer*****.  2. Actigraph accelerometer.  3. Pedometer. | Baseline, 3 (end of intervention), 9, and 15 months. |
| McMahon et al. 2017 | 102 (75.3) | 79 (6.5) | Inclusion:  Aged ≥ 70 years, speak English, ability to walk, no neurocognitive disorder, ≤ 21 telephone MMSE, PA level below national recommendations (strength <2times/week, M-V PA <150min/week). | Neighbourhood and home setting | 1. Self-report PA duration (average minutes of total physical activity in a 1-week period).  2. Physical function (gait speed, balance, lower-limb strength). | 1. Fitbit One Activity monitor and CHAMPS PA questionnaire*****.  2. SPPB | Baseline, 2 (end of intervention) and 8 months. |
| Uusi-Rasi et al. 2017 | 409 (100) | 74.1 (3) | Inclusion:  No contraindication for exercise, with a fall in the previous 12 months.  Exclusion:  Use of Vitamin D, M-V PA >2hours/week. | Exercise hall and Gymnasium | 1. Self-report PA level (duration) across domains, and of different intensities (light, moderate, vigorous) in a 1-week period.  2. Steps per day.  3. General mobility  4. Physical function (gait speed, balance, lower-limb strength).  5. Dynamic balance  6. Maximal isometric leg-extensor strength | 1. CHAMPS PA questionnaire*****.  2. Pedometer.  3. Timed Up and Go Test.  4. SPPB  5. Backwards walking test.  6. Strain gauge dynamometer. | Baseline, 24 (end of intervention), 36 and 48 months. |
| Martin-Borràs et al. 2018 | 422 (60.1) | IG: 69.5 (8.4)  CG: 68.2 (8.9) | Inclusion:  Participants of both genders aged 18–85, with at least one chronic disease, independent in rising from a chair and walking with or without a technical aid, self-reported being insufficiently active as determined by one question screening tool.  Exclusion:  Had a diagnosis of severe dementia (not able to understand and/or follow verbal commands), or had had a stroke, hip fracture, myocardial infarction or had undergone hip or knee replacement surgery within the previous 6 months. | Primary Care setting/ outdoors (e.g. public parks) | 1. Self-report PA (number of metabolic equivalents × minutes of activity × events per week).  attitude towards the  2. PA practice stage of change using the Prochaska scale.  3. Social support for PA practice. | 1. IPAQ short version*****.  2. Stages of change.  3. Social Support for Physical Activity Scale. | Baseline, 3 (end of intervention), 9 and 15 months. |

IG: Intervention Group; CG: Control Group; PA: Physical Activity; TGUT: Timed Get Up and Go Test; MMSE: Mini Mental State Examination; RES: Progressive resistance training; BAL: Balance jumping training; COMB: combination of RES+BAL trainings; MET: Metabolic Equivalents; SPPB: Short Physical Performance Battery; CHAMPS: Community Healthy Activities Model Program for Seniors; NZ: New Zealand; PT: Physical Therapist; LASA: Longitudinal Aging Study Amsterdam; M-V: moderate-vigorous; IPAQ: International Physical Activity Questionnaire.
