# Supplementary material for: Exercise-Based Interventions to Enhance Long-Term Sustainability of Physical Activity in Older Adults: A Systematic Review and Meta-Analysis of Randomized Clinical Trials
Source: Int J Environ Res Public Health. 2019 Jul 15;16(14):2527. doi: 10.3390/ijerph16142527 (PMC6678490; doi:10.3390/ijerph16142527)
Supplement: Supplementary file 1 [file ijerph-16-02527-s001.zip › ijerph-528093/Supplementary_Table_S4.docx]

Supplementary Table S4. Description of the interventions and strategies to enhance long-term adherence of PA levels

| **Study** | **Intervention description** | **Control description** | **Frequency, duration and intensity** | **Compliance to intervention** | **Person delivering the intervention** | **Strategies to enhance long-term sustainability of PA levels** | **Strategies related to individual behavior change based on the Social Cognitive Theory** |
| --- | --- | --- | --- | --- | --- | --- | --- |
| Ståhle et al. 1999 | Aerobic outpatient group-training program.  The training was followed by 10 min of music-supported relaxation. | Verbal and written information about the importance of regular PA.  Recommendation to take a daily walk at a comfortable speed, and to gradually increase this effort as soon as they felt able to do so.  Invitation to monthly information meetings at the department. | 12 weeks (3 months).  3 times/week of 50 min sessions.  Music was used to guide the intensity of the performance during the sessions. | Average compliance in IG: 87% (range 64-100). | Specialized physiotherapist. | IG: After the initial 12 weeks, patients were offered to participate in the program once a week for another 12 weeks.  CG: After the 12-week follow-up, participants were encouraged to contact the local National Association for Heart and Lung Patients to take part in its training program. | Behavior Capability  (Behavioral Strategy) |
| Hauer et al. 2003 | High-intensity progressive resistance training of functionally relevant muscle groups and a progressive functional and balance training program. | Motor placebo activities such as stretching or playing ball games while in a predominantly sitting position. | IG: 12 weeks (3 months).  3 times/week. Intensity of strength training was adjusted between 70% and 90% of each individual’s maximal workload. Basic training in functions such as walking, stepping, or balancing was progressive and training tasks with increasing complexity were introduced in the training regimen and protocol.  CG: 12 weeks.  3 times/week of 60 min sessions.  Both groups received identical physiotherapy twice a week for 25 minutes. Strength and balance training was excluded during physiotherapy and control group sessions. | Drop out at baseline n=13.  IG: Drop out follow-up testing n=2.  CG: Drop out follow-up testing n=0.  No data on attendance. | Not reported. | Not reported. | Not reported. |
| Beyer et al. 2007 | Flexibility-warm up 15 min, resistance exercise 30 min, balance training 10 min, stretching 5 min. | Usual care.  No exercise-related activities were provided for the control group. | 24 weeks (6 months).  2 times/week of 60 min sessions.  The training program was personalized because of heterogeneity of participants regarding physical symptoms | Training compliance was on average 79% (42-100%).  IG drop out n=8.  CG drop out n=1. | Supervised by a physiotherapist. | Participants were given information about health benefits of being physically active.  Physiotherapists applied the principles of self-efficacy, regular performance feedback and positive reinforcement to enhance the motivation for exercise progression. | Self-efficacy  (Cognitive Strategy)  Expectancies  (Cognitive Strategy) |
| McAuley et al. 2007 | Walking program. | Stretching and toning program. | 24 weeks (6 months).  3 times/week (length of each session not reported). | Drop out follow up at year 5: 21%. | Trained exercise specialists. | Participants were paid $30 for completion of measures at both time points.  Researchers used self-efficacy as the guiding theoretical construct. Participants were sent their measures’ feedback in a postage-paid envelope. | Self-efficacy  (Cognitive Strategy)  Reinforcement |
| Witham et al. 2007 | Progressive, seated exercise using small wrist and ankle weights to augment the aerobic component of the exercise. | Usual care.  No exercise-related activities were provided for the control group. | 12 weeks (3 months).  2 times/week (length of each session not reported). | Not reported. | Supervised by a physiotherapist. | Patients kept an activity diary and agreed to weekly goals for increased activity with the physiotherapist.  After the initial 3-month phase, patients in the exercise group continued to exercise at home for an additional 3 months with weekly telephone contact by the physiotherapist. | Self-efficacy  (Cognitive Strategy)  Self-control (activity diary and weekly goals)  (Behavioral Strategy) |
| Karinkanta et al. 2009 | Progressive Resistance Training (RES): exercises for large muscle groups.  Balance jumping training (BAL): modified aerobics and step aerobics, including a variety of balance, agility, and impact exercises.  RES+BAL (COMB). | Usual care.  No exercise-related activities were provided for the control group.  Participants were asked to maintain their pre-study level of PA during the 12-month trial. | 12 months.  3 times/week (length of each session not reported).  RES: intensity increased from 50-60% of 1RM to 75-80% of  1RM.  BAL: the degree of difficulty of movements, steps, impacts and jumps was gradually increased. | Training compliance was 74.4 (23.1) in group RES; 59.2 (29.3) in group BAL; and 67 (24.8) in group COMB. | Trained exercise  leaders. | Not reported. | Not reported. |
| Rejeski et al. 2009 | Aerobic walking exercise, along with strength, balance, and flexibility training. | Active control group with health education workshops on a variety of health topics relevant to older adults and also involved a short instructor-led program (5-10 minutes) of upper extremity stretching exercises. | 12 months.  IG: the intervention was divided into three phases: adoption (weeks 1-8) with 3 center-based exercise sessions (40–60 min) per week; transition (weeks 9-24) with 2 sessions/week and home-based endurance/ strengthening/ flexibility exercises (≥3/week); and maintenance (week 25 to the end of the trial) with home-based intervention, optional once-to-twice-per-week center-based sessions, and monthly telephone contacts.  During the first 12 weeks the PA intervention focused on walking. The goal was walking for at least 150 minutes over the course of the week, attained in a progressive and individualized manner. Participants also completed lower extremity strengthening and stretching exercises. Balance training was introduced during the adoption phase. The intensity of training was gradually increased over the first 2-3 weeks. Perceived exertion assessed by the Borg scale was used to regulate the intensity of exercise; moderate intensity exercise was promoted.  CG: participants met in small groups once/week for the first 26 weeks and then monthly. | Not reported. | Not reported. | Each participant received a 45-minute individualized, introductory session to describe the intervention and to provide individual counselling to optimize safety and participation. Once a week for the first 10 weeks, participants engaged in group mediated behavioral counselling sessions that focused on self-regulatory skills central to promoting PA and on the role of PA in disability prevention. | Self-efficacy  (Cognitive Strategy)  Behavioral Capability  (Behavioral Strategy) |
| Patel et al. 2013 | Green prescription* pedometer-based.  Green prescription traditional time-based.  Both options with 3 counselling calls, and daily logs. | Time-based goals for PA, plus 3 phone calls. | 12 weeks (3 months).  No frequency or duration was provided in the present article or the two previous papers^49,50^. | N=330 started, n=270 completed.  This study used the first 225 that completed. | Patient-support counsellor. | 1 phone call/month during the 12 weeks of intervention to counsel PA practice.  Physician prescribed PA.  Step and time-based goals were individualized and encouraged to increase over the course of time. | Self-efficacy  (Cognitive strategy)  Self-control  (Behavioral Strategy) |
| Dohrn et al. 2017 | Balance and walking. There were 2 intervention groups, one did balance training only and the other did balance training + Nordic walking but both groups were collapsed for data analysis. | Usual care.  No exercise-related activities were provided for the control group.  Participants were asked to continue with their usual activity.  The control group was offered the balance training following the 12-week period. | 12 weeks (3 months).  3 times/week of 45 minutes’ sessions | Dropout rate: 42%.  Only those who completed 24 sessions or more were included. | 2-3 Physiotherapists | Not reported. | Not reported. |
| McMahon et al. 2017 | 3 intervention groups:  OTAGO+PA monitor+ interpersonal behavioral strategies.  OTAGO+PA monitor+ intrapersonal behavioral strategies.  OTAGO+PA monitor+ interpersonal behavioral strategies+ intrapersonal behavioral strategies. | OTAGO+PA monitor | 8 weeks’ duration, 90min/week.  Small groups (4-6 participants). | With Fitbit One and Intervention workbook  End of intervention: 95%.  6 month FU: 93%.  Control: 6.9 meetings of 8. | Board-certified gerontological nurse practitioner. | Participants were given thank you cards and $20 following each of the three data collection sessions.  They were also invited to keep their physical activity monitors after the study to control their activity. | Reinforcement  (Behavioral Strategy)  Self-control  (Behavioral Strategy) |
| Uusi-Rasi et al. 2017 | 2 intervention groups:  VitD with Exercise.  No VitD with Exercise.  Exercise: strength large muscle groups, balance, agility and mobility. | 2 control groups:  No Exercise with VitD.  No exercise without VitD.  Participants were asked to continue with their usual activity. | 2 years intervention:  0-12 months: 2 times/week of 60 minutes sessions (10 min warm-up and stretching).  12-24 months: 1 time/week + house exercising.  Training sessions carried out in 8 week periods in groups of 10-20 participants.  1^st^ year intervention: home program the days they don’t participate in supervised sessions.  2^nd^ year intervention: home training sessions at least 3 times a week.  One home session requires 5-10 minutes.  Exercise intensity was individually progressive, estimated in metabolic equivalent tasks every 8  weeks with heart rate monitor (Firstbeat technologies). | Not reported | Supervised by a physiotherapist. | After the intervention, participants were followed for two years, and during this time period they continued to keep the falls diaries and pedometers.  In addition, all women were invited to continue with the exercise training, or to participate in any other exercise training they preferred. | Self-control (pedometers and fall diaries)  (Behavioral Strategy)  Behavior Capability  (Behavioral Strategy) |
| Martin-Borràs et al. 2018 | Primary care based ERS linked to municipal resources and social support and social participation enhancement.  Sessions included aerobic activities, upper and lower body strength-based exercises and balance exercises. | Usual care.  No exercise-related activities were provided for the control group.  Participants were asked to continue with their usual activity. | 12 weeks (3 months).  2 times/week of 60 minutes’ sessions.  Groups of 10–15 participants.  Participants were instructed to perform strength training at a perceived exertion intensity of 4–6 (somewhat hard) during the first 2 weeks of training. Training intensity was individually calculated by the 4–8 repetition maximum method for each exercise using elastic bands and body weight (loaded sit to stand movement). After the familiarization stage, preferred intensity was established at 6–8 (hard to really hard) of the Borg’s scale. | Compliance (attendance)  156/220 participants in the IG attended ≥19 of the total sessions. There were no adverse events during the study period. | Trained exercise specialists. | The exercise specialist encouraged all participants to conduct a third session each week on their own such as brisk walking to enhance the autonomy of the participants. The PA specialist detected a leader in each group to organize these extra sessions.  All participants were offered a personalized exercise program with exercises that were performed during the sessions.  During the last two sessions, visits with all participants were made to the nearest community resources (e.g., sport facilities) where the regular PA practice could be continued.  The PA program included the following mechanisms to enhance social support during the cool-down phase of each session: social influence/social comparison, social control, self-esteem, sense of control and belonging and companionship. | Self-efficacy  (Cognitive Strategy)  Behavior Capability (Behavioral Strategy)  Expectations  (Cognitive Strategy) |

*Green Prescription is a prescription for PA administered by a physician in the same format that drug treatment is administered: type, intensity, and frequency.

PA: Physical Activity; IG: Intervention Group; CG: Control Group; RM: Repetition Maximum; FU: Follow Up; ERS: Exercise referral Scheme.
