# Supplementary figures and images for: Exercise-Based Interventions to Enhance Long-Term Sustainability of Physical Activity in Older Adults: A Systematic Review and Meta-Analysis of Randomized Clinical Trials
Source: Int J Environ Res Public Health. 2019 Jul 15;16(14):2527. doi: 10.3390/ijerph16142527 (PMC6678490; doi:10.3390/ijerph16142527)

**Supplementary Table S2**


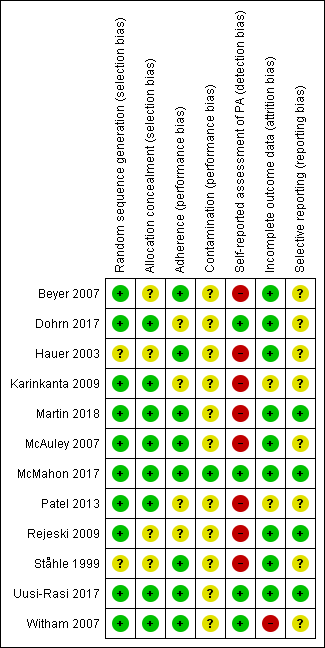

Supplement: Supplementary file 1 [file ijerph-16-02527-s001.zip › ijerph-528093/Supplementary_Table_S2_Risk_of_bias.docx]
